# Dyslexia‐Related Hearing Loss Occurs Mainly through the Abnormal Spontaneous Electrical Activity of Spiral Ganglion Neurons

**DOI:** 10.1002/advs.202205754

**Published:** 2023-04-17

**Authors:** Guodong Hong, Xiaolong Fu, Xin Chen, Liyan Zhang, Xuan Han, Shuqin Ding, Ziyi Liu, Xiuli Bi, Wen Li, Miao Chang, Ruifeng Qiao, Siwei Guo, Hailong Tu, Renjie Chai

**Affiliations:** ^1^ State Key Laboratory of Bioelectronics Department of Otolaryngology Head and Neck Surgery Zhongda Hospital School of Life Sciences and Technology Advanced Institute for Life and Health Jiangsu Province High‐Tech Key Laboratory for Bio‐Medical Research Southeast University 210096 Nanjing China; ^2^ Co‐Innovation Center of Neuroregeneration Nantong University 226001 Nantong China; ^3^ Medical Science and Technology Innovation Center Shandong First Medical University & Shandong Academy of Medical Sciences 250000 Jinan China; ^4^ Department of Otolaryngology Head and Neck Surgery Sichuan Provincial People's Hospital University of Electronic Science and Technology of China 610072 Chengdu China; ^5^ Institute for Stem Cell and Regeneration Chinese Academy of Science 100101 Beijing China; ^6^ Beijing Key Laboratory of Neural Regeneration and Repair Capital Medical University 100069 Beijing China; ^7^ School of Life Science Shandong University 266237 Qingdao China

**Keywords:** Dyslexia, *Dyx1c1*, hearing loss, neurodevelopmental disorder

## Abstract

Dyslexia is a reading and spelling disorder due to neurodevelopmental abnormalities and is occasionally found to be accompanied by hearing loss, but the reason for the associated deafness remains unclear. This study finds that knockout of the dyslexia susceptibility 1 candidate 1 gene (*Dyx1c1^−/−^*) in mice, the best gene for studying dyslexia, causes severe hearing loss, and thus it is a good model for studying the mechanism of dyslexia‐related hearing loss (DRHL). This work finds that the *Dyx1c1* gene is highly expressed in the mouse cochlea and that the spontaneous electrical activity of inner hair cells and type I spiral ganglion neurons is altered in the cochleae of *Dyx1c1^−/−^* mice. In addition, primary ciliary dyskinesia‐related phenotypes such as situs inversus and disrupted ciliary structure are seen in *Dyx1c1^−/−^* mice. In conclusion, this study gives new insights into the mechanism of DRHL in detail and suggests that *Dyx1c1* may serve as a potential target for the clinical diagnosis of DRHL.

## Graphical abstract

Compared with wild‐type mice, the process of type I spiral ganglion neuron (SGN) refinement and signaling transduction between the inner hair cell (IHC) and type I SGN is disrupted in *Dyx1c1^–/–^* mice (Bottom left panel). Moreover, *Dyx1c1* deficiency damaged the kinociliary structure and planar cell polarity (PCP) of hair bundle (Bottom left panel).

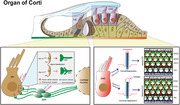

## 1 Introduction

Hearing loss is a complex disease resulting from many factors such as heredity, mechanical damage, age, noise, and ototoxic drugs. The number of deafness patients worldwide has reached 466 million, and it will continue to increase and is expected to reach 900 million in 2050.^[^ ^1^ ^]^ Cochlear hair cells (HCs) are important mechanoreceptors in the auditory system and can convert mechanical sound signals into electrical signals, which are then transmitted by spiral ganglion neurons (SGNs) to the auditory cortex for information processing, and defects at any step can lead to severe hearing loss.^[^ ^2^, ^3^, ^4^ ^]^ At present, determining the pathogenesis of deafness in various human diseases remains a serious challenge.

Dyslexia is a common learning disability characterized by auditory/phonological deficits, and even though patients have normal intelligence and educational opportunities, they are inferior to normally developing children in reading and phonological comprehension.^[^ ^5^ ^]^ Dyslexia cannot be explained simply by a genetic disorder. It is currently thought to be caused by the interaction of environment and genetics, with the genetic component accounting for up to 60% of the disease.^[^ ^6^ ^]^ About 5% –18% of the world's people suffer from varying degrees of dyslexia, therefore, a comprehensive analysis of dyslexia characteristics is essential for the treatment of patients.^[^ ^7^, ^8^ ^]^ The clinical symptoms of dyslexia mainly include defects in speech processing,^[^ ^9^, ^10^, ^11^ ^]^ short‐term working memory,^[^ ^12^, ^13^, ^14^ ^]^ and rapid auditory processing,^[^ ^15^, ^16^, ^17^ ^]^ which provide a theoretical basis for the pathogenesis of dyslexia. According to previous reports, a novel intronic single nucleotide variant and three novel intergenic single nucleotide variants in the broad region of the human roundabout guidance receptor 1 (*ROBO1*) gene cause dyslexia, and patients with *ROBO1* mutation show deficits in phonological awareness, short‐term memory, and auditory processing.^[^ ^18^ ^]^ Subsequently, doublecortin domain containing 2 (*DCDC2*) gene was found to be expressed in the brain region where fluent reading occurs and that *DCDC2* gene mutations disrupt neuronal development and lead to a series of dyslexia symptoms.^[^ ^19^ ^]^ In addition, four of the 18 single nucleotide polymorphisms (SNPS) in *KIAA0319* (Kazusa DNA Research Institute, KI; reference characters, AA; 0319) were significantly associated with dyslexia.^[^ ^20^ ^]^ Taken together, these studies showed that dyslexia is a complex clinical syndrome associated with multiple pathological conditions, including neurodevelopmental disorders.

Patients with dyslexia can show abnormal auditory brainstem response (ABR).^[^ ^21^, ^22^ ^]^ Scientists previously recorded early evoked ABRs to 500 Hz tone bursts in five dyslexic patients, and the results showed that the dyslexic patients exhibited aberrant early evoked response waveforms. These dyslexic patients had undetectable ABR waveforms even at intense stimulation levels (sound‐pressure level (SPL) of 70 dB), indicating severe hearing loss.^[^ ^21^ ^]^ However, some investigators examined ABR thresholds in 24 dyslexic patients who were completely unable to read and write, but did not find any evidence of auditory brainstem dysfunction.^[^ ^23^, ^24^ ^]^ These divergent conclusions seem to be explained by genetic heterogeneity, and thus the etiology of dyslexia‐related hearing loss (DRHL) requires further exploration.

Researchers have performed acoustic stimulation on 20 dyslexic children to test whether the subjects responded correctly to sound signals, and there was no difference between patients and normal subjects when the sound stimuli were given at a slow rate, but when the interval between consecutive stimuli was shortened the dyslexic patients showed more false responses, suggesting that dyslexic patients have deficits in rapid auditory processing.^[^ ^25^ ^]^ Subsequently, rapid auditory processing was examined in 10 adults with dyslexia and 20 control subjects using short click intervals and found that the adults with dyslexia had deficits in rapid auditory processing.^[^ ^26^ ^]^ Until now, it has been unclear whether DRHL is related to the abnormal rapid auditory processing associated with dyslexia. Although people have long paid attention to the relationship between dyslexia and hearing, there are no reports on the characteristics and mechanisms of DRHL in dyslexic patients, so there is an urgent need to explore the mechanism of deafness in dyslexic patients.

The first dyslexia susceptibility gene *Dyx1c1* located near the *Dyx1* locus on chromosome 15q21 and that the t (2;15) (q11; q21) translocation segregates simultaneously with dyslexia.^[^ ^8^ ^]^ At present, the *Dyx1c1^−/−^* mouse is an ideal model of dyslexia and is widely used to study the pathogenesis of dyslexia‐related deficits.^[^ ^27^, ^28^, ^29^, ^30^, ^31^ ^]^ Knockdown of *Dyx1c1* in rat embryos leads to the loss of the ability of the neocortical neurons to migrate to the proper position, indicating that *Dyx1c1* is essential for nervous system development.^[^ ^32^ ^]^ Further studies showed that *Dyx1c1* knockdown in the forebrain of the mouse resulted in impairment of learning and memory, indicating that *Dyx1c1* is critical for the development of the learning and memory part of the brain.^[^ ^33^ ^]^ Another study found that neurons failed to migrate after *Dyx1c1* deficiency, which in turn led to disruption of auditory processing.^[^ ^34^ ^]^ In addition, *Dyx1c1* deficiency also causes primary ciliary dyskinesia (PCD).^[^ ^35^, ^36^, ^37^ ^]^ *Dyx1c1* is expressed in the cytoplasm of respiratory epithelial cells and involved in the assembly of ciliary dynein, and *Dyx1c1^−/−^* mice presented with phenotypes such as ciliary immobility and situs inversus similar to PCD, suggesting that *Dyx1c1* is a novel axonal dynein assembly factor.^[^ ^35^ ^]^ Coincidentally, patients with PCD are often accompanied by auditory dysfunction.^[^ ^38^, ^39^ ^]^ The interesting thing is that the dyslexia susceptibility genes *ROBO1*, *DCDC2*, and *KIAA0319* are all involved in ciliary function.^[^ ^18^, ^40^, ^41^ ^]^ In this study, we found that knockout of *Dyx1c1* caused severe deafness in mice, therefore, as a key susceptibility gene for dyslexia, *Dyx1c1* appears to be a key factor linking hearing impairment with dyslexia.

In this study, we established *Dyx1c1^−/−^* mice to explore the mechanism of DRHL. We found that *Dyx1c1* was strongly expressed in the mouse cochlea, and the *Dyx1c1^−/−^* mice exhibited severe deafness. Further experiments showed that the structure of the kinocilia and the developmental type I SGNs was abnormal in cochlea of *Dyx1c1^−/−^* mice, which cause severe hearing loss. These findings will help us better understand the characteristics and roles of *Dyx1c1* functional deficits in hearing loss and provide new insights into the etiology and treatment of DRHL.

## 2 Results

### 2.1 *Dyx1c1* is Highly Expressed in the Mouse Cochlea

**Figure 1:**
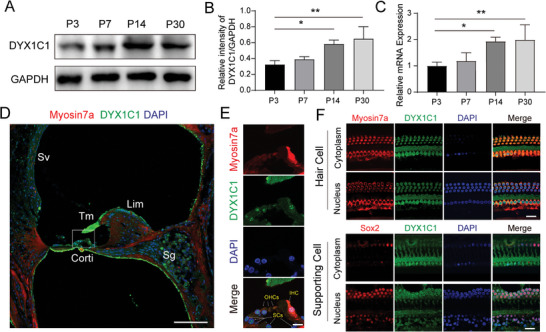
*Dyx1c1* is highly expressed in the mouse cochlea. A) Western blots of protein lysates of the whole cochlea show increasing *Dyx1c1* protein level from P3 to P30. B) Quantification of *Dyx1c1* protein levels in (A); *n* = 3 for each group. C) qPCR analysis of mRNA of the whole cochlea shows increasing *Dyx1c1* mRNA levels from P3 to P30; *n* = 3 for each group. D) Immunofluorescence staining of cochlear cryosections from P30 WT mice revealed that *Dyx1c1* was expressed in HCs, SCs, and SGNs. Immunofluorescence staining of cochlear cryosections of P30 WT mice with anti‐Myosin7a (red), anti‐*Dyx1c1* (green), and DAPI (blue) antibodies. Scale bar = 100 µm. Sv, stria vascularis; Lim, spiral limbus; Tm, tectorial membrane; Sg, spiral ganglion. E) Enlarged view of the organ of Corti in (D). Scale bar = 10 µm. F) The whole cochleae of P30 WT mice were used for immunofluorescence experiments. HCs, SCs, *Dyx1c1* protein, and nuclei were labeled with anti‐Myosin7a (red), anti‐Sox2 (red), anti‐ *Dyx1c1* (green), and DAPI (blue), respectively. The results showed that *Dyx1c1* was expressed in the cytoplasm and nuclei of HCs and SCs. Scale bar = 10 µm. Data are shown as the mean ± S.D. *p < 0.05, **p < 0.01 using two‐tailed, unpaired Student's *t*‐tests.

First, we extracted protein lysates from the cochleae of wild‐type (WT) mice at postnatal day (P)3, P7, P14, and P30 for immunoblotting experiments, and found that the expression of *Dyx1c1* gradually increased in the mouse cochlea after birth, which was confirmed by subsequent qPCR experiments (**Figure** **1**A–C). Next, the subcellular localization of *Dyx1c1* in the mouse cochlea was determined using immunofluorescence. We found that *Dyx1c1* was highly expressed in the SGNs, tectorial membrane, the organ of Corti, and the spiral limbus at P30 (Figure 1D). In addition, *Dyx1c1* was localized in the nucleus and cytoplasm of hair cells (HCs) and supporting cells (SCs) in the organ of Corti, and the fluorescence intensity seemed to indicate that *Dyx1c1* was expressed more in HCs than in SCs (Figure 1E). Subsequently, we further observed the subcellular localization of *Dyx1c1* in the organ of Corti using the whole basement membrane technique. Similarly, the *Dyx1c1* was localized in both the nucleus and cytoplasm of SCs (labeled with anti‐Sox2 antibody) and HCs (Figure 1F). The extensive expression of *Dyx1c1* in the cochlea suggests that it might be critical for auditory function.

### 2.2 *Dyx1c1^−/−^* Mice Suffered from Severe Hearing Loss in Addition to Dyslexia‐Related Deficits

**Figure 2:**
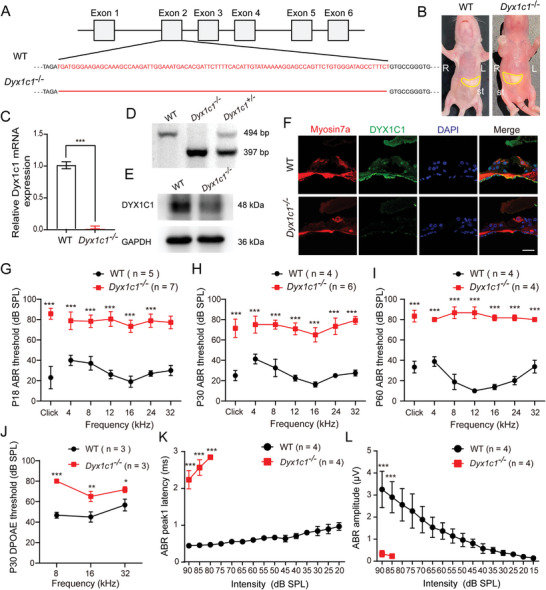
*Dyx1c1^−/−^* mice suffer from severe hearing loss. A) *Dyx1c1^−/−^* mice were constructed by deleting a specific sequence in the exon 2 region of the *Dyx1c1* gene using CRISPR‐Cas9 technology. B) The milk‐filled stomach is inverted to the right in *Dyx1c1^−/−^* mice. The yellow area represents the location of the stomach. St, stomach; R, right; L, left. C) qPCR results showed that *Dyx1c1* mRNA was significantly decreased in the cochleae of *Dyx1c1^−/−^* mice. D) PCR indicating the genotyping results from the WT, *Dyx1c1^−/−^*, and *Dyx1c1^+/−^* mice. E) Western blotting showed that the presence of Dyx1c1 protein was detected in the WT mouse cochlea, but was absent in *Dyx1c1^−/−^* mice. F) The mouse cochlea was co‐stained with anti‐Myosin7a antibody (red), anti‐Dyx1c1 antibody (green), and DAPI (blue). The *Dyx1c1* fluorescence signal was distributed in the organ of Corti in WT mice, but almost no fluorescence signal was seen in *Dyx1c1^−/−^* mice. Scale bar = 20 µm. G–I) P18, P30, and P60 *Dyx1c1^−/−^* mice had increased click and tone‐burst ABR thresholds between 4 and 32 kHz compared to WT mice. J) The DPOAE thresholds of *Dyx1c1^−/−^* mice at P30 were significantly increased at 8, 16, and 32 kHz compared to WT mice. *n* = 3 for each group. K–L) Statistical analysis showed that both the latency and amplitude values of peak 1 in *Dyx1c1^−/−^* mice were 2‐ to 3‐fold higher than those in WT mice. Data are shown as the mean ± S.D. *p < 0.05, **p < 0.01, ***p < 0.001 using two‐tailed, unpaired Student's *t*‐tests.

To determine the biological function of *Dyx1c1* in the auditory system, we inactivated *Dyx1c1* by deleting a region in exon 2 of the gene using CRISPR‐Cas9 technology (**Figure** **2**A). Consistent with previous reports, *Dyx1c1^−/−^* mice had similar defects as those observed in the dyslexia mice, such as situs inversus (Figure 2B). The percentage of *Dyx1c1^−/−^* mice produced by heterozygous mice was exceedingly low, and only a few knockout mice survived. In addition, *Dyx1c1^+/−^* mice were viable, and there was no significant difference compared to WT mice (data not shown). We next confirmed that *Dyx1c1* was knocked out in the cochleae of *Dyx1c1^−/−^* mice by qPCR, PCR, and western blot experiments (Figure 2C–E), and immunofluorescence experiments showed that the fluorescent signal of *Dyx1c1* protein was significantly reduced in the cochleae of *Dyx1c1^−/−^* mice (Figure 2F). To further explore whether the *Dyx1c1^−/−^* mice have dyslexia‐related phenotype, we performed novel object recognition (NOR) test in P30 WT and *Dyx1c1^−/−^* mice, the results showed that the *Dyx1c1^−/−^* mice had a much lower ability to recognize novel objects than the WT mice (Figure S1A, Supporting Information). These results indicate that the *Dyx1c1^−/−^* mice had been successfully engineered and could be used to explore the correlation between dyslexia and hearing loss.

We next assessed the auditory function of *Dyx1c1^−/−^* mice using the click and tone‐burst auditory brainstem response (ABR) technique. The ABR thresholds for click stimuli and tone‐burst between 4 and 32 kHz at P18, P30, and P60 *Dyx1c1^−/−^* mice were significantly increased by ≈40 to 60 dB sound pressure level (SPL) compared to WT mice (Figure 2G–I). Distortion product otoacoustic emissions (DPOAE) was subsequently measured and the thresholds in *Dyx1c1^−/−^* mice were significantly higher than WT mice, indicating impaired function of outer hair cells (Figure 2J). Next, we analyzed the ABR peak 1 latency and amplitude values at 16 kHz, and the ABR threshold of *Dyx1c1^−/−^* mice was only about 80 dB (Figure S1B, Supporting Information), and the peak 1 latency and amplitude were both 2 to 3 times greater than in WT mice (Figure 2K,L), which suggests that the function of the SGN in the cochleae of *Dyx1c1^−/−^* mice is disrupted. Taken together, these results demonstrate that knockout of *Dyx1c1* causes severe deafness.

### 2.3 No Significant Changes were Seen in the Cochlear Structure in *Dyx1c1^−/−^* Mice

**Figure 3:**
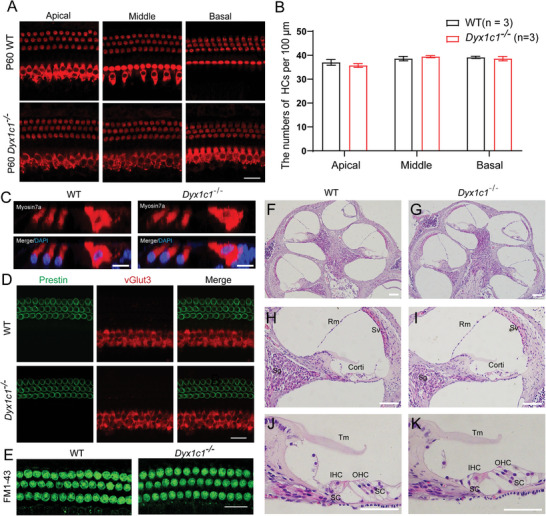
*Dyx1c1^−/−^* mice have normal cochlear structure. A–C) The morphology and number of HCs in *Dyx1c1^−/−^* mice were comparable to those in WT mice at P60. HCs were labeled with anti‐Myosin7a antibody (red), scale bar = 20 µm. B) The number of HCs in (A), *n* = 3 for each group. C) Normal morphology of hair cells was found between in WT and *Dyx1c1^−/−^* mice cochleae. Hair cells and nuclei were labeled with anti‐Myosin7a (red) and DPAI (blue) antibodies, respectively. Scale bar = 10 µm. D) IHCs and OHCs were labeled with anti‐vGlut3 (red) and anti‐Prestin antibodies (green), respectively. Scale bar = 20 µm. E) FM1‐43 staining showed that the MET function of hair bundles in the *Dyx1c1^−/−^* mouse cochlea was comparable to that in WT mice. Scale bar = 20 µm. F–K) *H&E* staining analysis of the P30 WT (F,H,J) and *Dyx1c1* knockout cochlea (G,I,K). No significant difference was found in the morphology of the organ of Corti, stria vascularis (Sv), reissner's membrane (Rm), tectorial membrane (Tm), and spiral ganglion (Sg) of the cochlea between the WT and *Dyx1c1^−/−^* mice. Scale bar = 100 µm.

Dysfunction of HCs in the inner ear is the main cause of hearing loss, therefore, we systematically analyzed the cochlear structure of *Dyx1c1^−/−^* mice. The cochlear basement membrane of P60 mice was stained with an anti‐Myosin7a antibody to label HCs (red). Immunostaining results showed that the number and morphology of HCs in *Dyx1c1^−/−^* mice did not show any significant difference compared to WT mice (**Figure** **3**A–C). Next, we examined the localization of critical functional proteins in the inner HCs (IHCs) and outer HCs (OHCs), and immunofluorescence showed normal localization of the OHC marker Prestin and the IHC marker vGlut3 (Figure 3D). FM1‐43 is a styrene membrane dye that can be rapidly taken up by HCs and is widely used to reflect functional mechanoelectrical transduction (MET) channels in HCs. We rapidly dissected the cochleae from P18 mice and showed that the uptake of FM1‐43 dye by HCs in *Dyx1c1^−/−^* mice was very similar to WT mice, which suggests that the *Dyx1c1* gene is dispensable for the MET function of HCs (Figure 3E). *Dyx1c1* is widely expressed in the mouse cochlea, and it is necessary to analyze the structure of the cochlea in *Dyx1c1^−/−^* mice. *H&E* staining showed that the overall morphology of the cochlea in *Dyx1c1^−/−^* mice was similar to WT mice (Figure 3F,G), and no significant difference was found in the organ of Corti, spiral ganglion, tectorial membrane, and stria vascular (Figure 3H–K). *Dyx1c1* deficiency may not affect the structure of the cochlea. These results indicated that *Dyx1c1* knockout did not alter the MET function of hair bundles and the expression of core functional proteins in HCs.

### 2.4 Abnormal Degeneration of the Cochlear Kinocilium and Microvilli in *Dyx1c1^−/−^* Mice

**Figure 4:**
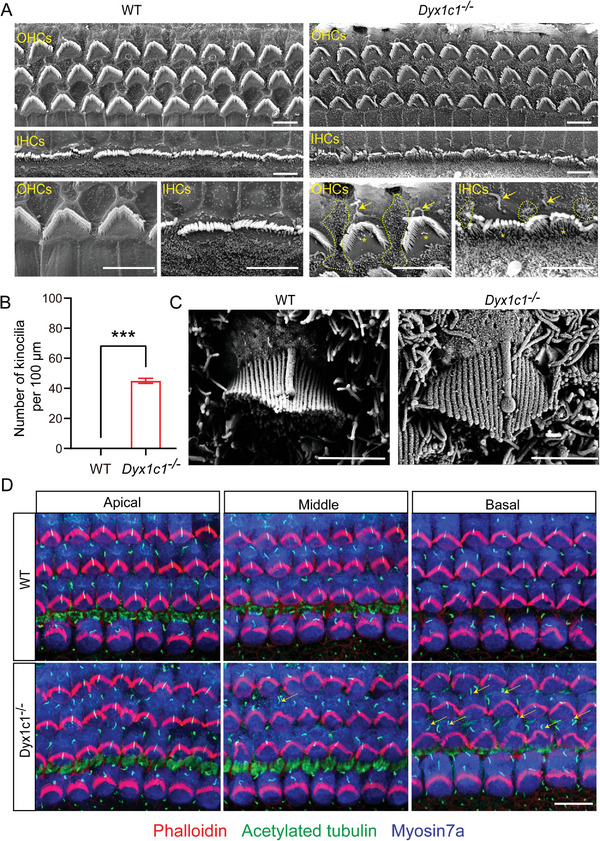
*Dyx1c1^−/−^* mice have defective kinocilia and microvilli in the cochlea. A) Representative SEM images of hair bundles in the middle turn of the cochlea from WT and *Dyx1c1^−/−^* mice at P18. Arrows represent kinocilia, while the asterisks and the dotted lines represent microvilli that were not degenerated in HCs and SCs, respectively. Scale bar = 5 µm. B) Statistical analysis of the number of kinocilia in (A). C) Representative SEM images of the kinocilium in the middle turn of the cochlea from WT and *Dyx1c1^−/−^* mice at P4. The tips of the kinocilia in the cochlea of *Dyx1c1^−/−^* mice were enlarged compare to WT. Scale bar = 2 µm. D) Immunofluorescence staining of the cochlear basement membrane of P4 mice. HCs, kinocilia, and hair bundles were labeled with anti‐Myosin7a (blue), anti‐acetylated tubulin (green), and phalloidin (red) antibodies, respectively. Scale bar = 10 µm. Data are shown as the mean ± S.D. ***p < 0.001 using two‐tailed, unpaired Student's *t*‐tests.

Hair bundles play an indispensable role in the process of auditory signal transduction in the cochlea, and irreversible damage to the hair bundles can lead to severe hearing impairment. We previously found that *Dyx1c1^−/−^* mice exhibited severe hearing loss from P18 to P60. To explore whether *Dyx1c1* deletion disrupts the structure of hair bundles in the cochlea, scanning electron microscopy (SEM) was performed. We found that numerous distorted kinocilia in the cochleae of *Dyx1c1^−/−^* mice were not completely degenerated compared to WT mice at P18, however, the shape and position of the hair bundles did not change significantly. In addition, there were large numbers of abnormal microvilli on HCs and SCs in the cochleae of *Dyx1c1^−/−^* mice (**Figure** **4**A,B). According to previous reports, the kinocilium degenerates prior to the activation of hearing in mice,^[^ ^42^ ^]^ and we used SEM to observe the structure of the kinocilium at P4. The distal ends of the kinocilia in the cochleae of *Dyx1c1^−/−^* mice were enlarged and presented as spheroids compared with the WT mice (Figure 4C). Subsequently, an immunofluorescence experiment was performed to further observe the distribution of kinocilia and hair bundles on HCs. In the basal turn of the cochleae of *Dyx1c1^−/−^* mice, many kinocilia deviated from the hair bundles, however, there was no significant difference in the orientation or morphology of the hair bundles between *Dyx1c1^−/−^* and WT mice (Figure 4D), which indicates that kinocilium integrity does not seem to be necessary for the orientation of the hair bundle.

Destruction of the kinocilia reduces their association with hair bundles and disrupts the planar cell polarity (PCP) of HCs, which in turn leads to severe hearing loss.^[^ ^43^ ^]^ To explore whether the PCP of HCs was disrupted in *Dyx1c1^−/−^* mice, we used an anti‐acetylated tubulin antibody to label kinocilia, a phalloidin antibody to label hair bundles, and an anti‐Myosin7a antibody to label HCs for immunostaining experiments. The orientation of the hair bundles of P0 *Dyx1c1^−/−^* mice deviated compared with WT mice, but this was unexpectedly corrected by P4 (Figure S2A, Supporting Information). The PCP of HCs depends on early differentiation and reorientation after birth, and the vertices of all hair bundles are uniformly aligned to point toward the outer (distal/lateral/non‐neural) edge of the cochlear helix, thus maintaining the correct orientation to respond to mechanical forces along the neuro‐abneural axis,^[^ ^44^ ^]^ we aligned the coordinate axis with the neural‐abneural axis for statistical hair bundle deflection analysis (Figure S2B,C, Supporting Information). The number of HCs used for statistical analysis exceeded 600 in both WT and *Dyx1c1^−/−^* mice for each age, and the results showed that the hair bundle orientation of the apical and middle turn was severely impaired in P0 *Dyx1c1^−/−^* mice (Figure S2D,E, Supporting Information). Surprisingly, this PCP defect was corrected at P4 despite the separation of kinocilia from hair bundles (analytical data are not shown). These results demonstrate that *Dyx1c1* gene deficiency reduces the association between kinocilia and hair bundles, which in turn disrupts the orientation of hair bundles, but the mechanism for repairing this PCP defect after birth in *Dyx1c1^−/−^* mice remains to be further explored.

### 2.5 *Dyx1c1* Deficiency Disrupts the Intraflagellar Transport Pathway in the Kinocilium

**Figure 5:**
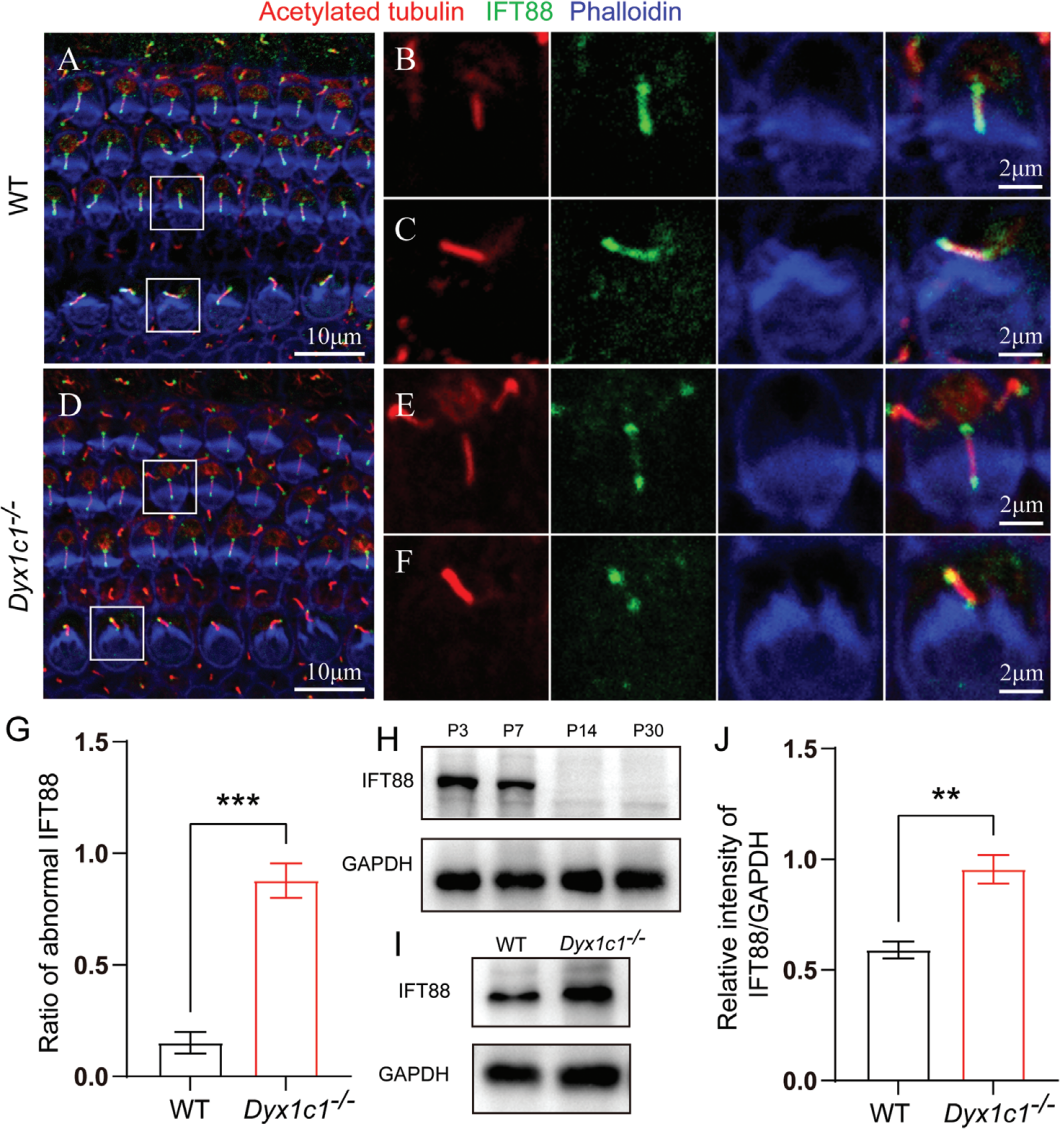
The IFT pathway is disrupted in the HCs of *Dyx1c1^−/−^* mice. Immunofluorescence images of cochlear epithelial cells in A–C) WT and D–F) *Dyx1c1^−/−^* mice. Hair bundles, kinocilia, and IFT88 protein were labeled with phalloidin (blue), anti‐acetylated tubulin (red), and anti‐IFT88 (green) antibodies, respectively. G) Statistical analysis of kinocilia with abnormal IFT88 levels in the cochleae of WT and *Dyx1c1^−/−^* mice; *n* = 3 for each group. H) Western blot showed that IFT88 was highly expressed at P3, decreased at P7, and was almost undetectable at P14 and P30. I) Western blot results indicated that the IFT88 protein level was higher in the cochleae of ​​P3 *Dyx1c1^−/−^* mice than in WT mice. J) Quantitative analysis of IFT88 in (I). Data are shown as the mean ± S.D. **p < 0.01, ***p < 0.001 using two‐tailed, unpaired Student's *t*‐tests.

Based on the previously described changes in ciliary structure, we speculated that the various components cannot be transported from the distal to the proximal end of kinocilium and cannot return to the cytoplasm of HCs in *Dyx1c1* ^−/−^ mice.^[^ ^45^, ^46^ ^]^ Cilia degeneration requires the involvement of the intraflagellar transport (IFT) pathway.^[^ ^46^ ^]^ To explore whether the altered kinociliary structure in *Dyx1c1^−/−^* mice is associated with an aberrant IFT pathway, we used immunostaining to observe the localization of intraflagellar transport 88 (IFT88)—which is a member of the IFT core subunits—and the results showed that IFT88 fluorescence signal was uniformly distributed from the proximal to distal ends of the kinocilia in WT mouse cochleae. However, the fluorescence signal of IFT88 was mainly enriched at the proximal and distal ends of the kinocilia of *Dyx1c1^−/−^* mice, and the IFT88 signals in the middle of the kinocilia were significantly weaker compared to WT mice (**Figure** **5**A–F). IFT88 was mislocalized in more than 60% of the kinocilia in the inner ear of *Dyx1c1^−/−^* mice (Figure 5G). Subsequently, western blot results showed that IFT88 was mainly expressed in the cochlea prior to the time of hearing onset (Figure 5H), and the IFT88 protein level in *Dyx1c1^−/−^* mice was higher than that in WT mice at P3 (Figure 5I,J). These results suggest that *Dyx1c1* deficiency blocked the IFT pathway and led to the failure of kinocilia degeneration.

### 2.6 Altered Spontaneous Activity of IHCs and SGNs in *Dyx1c1^−/−^* Mice

**Figure 6:**
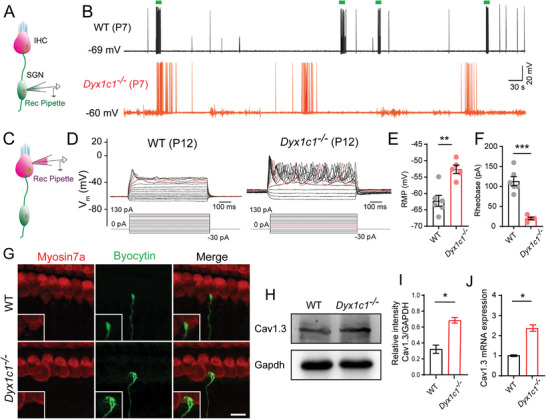
SGNs and IHCs in *Dyx1c1^−/−^* mice exhibit altered burst firing prior to hearing onset. A) Schematic of recording of spontaneous bursts of SGN action potentials. B) SGN spontaneous action potentials in *Dyx1c1^−/−^* and WT mice at P7. Green boxes in the WT group represent discrete action potential bursts, and no effective action potential bursts were seen in *Dyx1c1^−/−^* mice. C) Schematic for recording spontaneous bursts of IHC action potentials. D–F) HC spontaneous action potential results show that *Dyx1c1^−/−^* mice generated more action potentials (D), higher resting membrane potentials (E), and lower minimum amplitudes (F) required to elicit action potentials compared to WT mice. *n* = 6 IHCs for the WT group, *n* = 5 IHCs for the *Dyx1c1^−/−^* group. G) The recorded SGNs were injected with biocytin dye (green), and subsequent immunofluorescence showed more untrimmed branches at the ends of SGNs in *Dyx1c1^−/−^* mice compared to WT mice. HCs are labeled with anti‐Myosin7a antibody (red). Scale bar = 10 µm. H,I) Western blot showed that the protein level of *Cav1.3* in the *Dyx1c1^−/−^* cochlea at P12 was elevated compared with WT mice, and I) is the quantitative analysis of *Cav1.3* in (H). J) The qPCR results also showed that the mRNA level of *Cav1.3* in the cochlea of *​​Dyx1c1^−/−^* mice was higher than WT mice. Data are shown as mean ± S.D. *p < 0.05, **p < 0.01, ***p < 0.001 using two‐tailed, unpaired Student's *t*‐tests.

The kinocilia are lost prior to hearing onset, so it is thought that kinocilia are not directly involved in hearing.^[^ ^47^ ^]^ Although the hair bundles of *Dyx1c1^−/−^* mice were deficient in PCP at P0, PCP was self‐repaired at P4. Previous reports have suggested that slight alterations of kinocilia structure and PCP in the mouse cochlea do not cause severe hearing impairment; ^[^ ^48^, ^49^ ^]^ however, our previous ABR data showed that *Dyx1c1^−/−^* mice have extreme hearing impairment starting at P18. Therefore, we speculate that *Dyx1c1* deficiency may involve other factors leading to severe hearing loss in addition to the damaged kinocilium. Previous studies have shown that RNAi of *Dyx1c1* disrupts neuronal migration in the developing embryonic neocortex of rats.^[^ ^50^ ^]^ *Dyx1c1^−/−^* mice exhibited severe hearing loss, and the latency and amplitude of ABR peak 1 were significantly different from WT mice, suggesting a pathological defect in spiral ganglion neurons (SGNs). Next, we recorded the spontaneous activity of SGNs in WT and *Dyx1c1^−/−^* mice using the patch‐clamp technique at P7 and found that the number of effective discrete bursts of action potential in the SGNs of *Dyx1c1^−/−^* mice was smaller than WT mice, and the intensity of spontaneous activity was significantly reduced (**Figure** **6**A,B).

According to previous reports, continuous electrical signaling between IHCs and SGNs during the first two weeks of postnatal life in mice contributes to further refinement of the SGNs and solidifies the IHC‐SGN link.^[^ ^51^ ^]^ Therefore, we hypothesized that SGNs with weak spontaneous electrical activity might be caused by the lower spontaneous activity of IHCs. We recorded the spontaneous activity of IHCs in WT and *Dyx1c1^−/−^* mice using the patch‐clamp technique at P12 and found that the IHCs of *Dyx1c1^−/−^* mice generated more action potentials compared with WT mice (Figure 6C,D). The resting membrane potential and minimum magnitude of the current step required to elicit action potentials from IHCs were examined, and the IHCs from *Dyx1c1^−/−^* mice had higher resting membrane potentials and required lower currents (<50 pA) to elicit action potentials compared to WT mice (Figure 6E,F). The morphological features of the tested SGNs were subsequently examined by immunofluorescence according to the strategy of Alexander Markowitz,^[^ ^52^ ^]^ and this showed that the SGNs had redundant untrimmed branches (Figure 6G). The refinement of the terminal branches of SGNs at P7 was one of the characteristics of their maturation,^[^ ^53^, ^54^ ^]^ indicating that *Dyx1c1* deficiency hindered SGN development. Taken together, these results suggest that *Dyx1c1* deficiency severely reduces SGN and IHC maturation.

Cav1.3 is a voltage‐gated calcium channel that plays an important role in a variety of cellular functions in the inner ear, but it mainly mediates sustained Ca^2+^ influx and plays a role in HC development.^[^ ^55^ ^]^ Therefore, we compared the expression level of Cav1.3 in the inner ear of WT and *Dyx1c1^−/−^* mice at P12, and the western blot and qPCR results showed that the protein and mRNA levels of Cav1.3 were significantly increased in the cochleae of *Dyx1c1^−/−^* mice (Figure 6H–J), which was consistent with the enhanced spontaneous electrical activity of IHCs. These results implied that knockout of *Dyx1c1* perturbs the electrical signaling between IHCs and SGNs, thereby reducing the stability of the connection between IHCs and SGNs. This abnormal spontaneous electrical activity in the inner ear may be one of the key factors that lead to developmental disorders of the auditory system and thus to DRHL.

### 2.7 Structural Analysis of Ribbon Synapses in *Dyx1c1^−/−^* Mice

**Figure 7:**
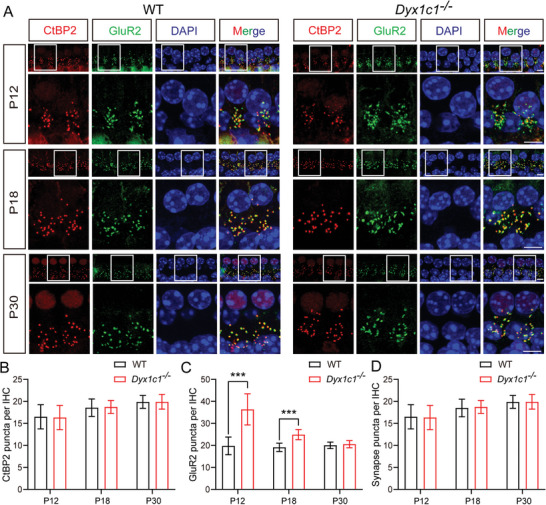
Abnormal development of ribbon synapses in the cochleae of *Dyx1c1^−/−^* mice. A) Staining of ribbon synapse structures in the mouse cochlea at P12, P18, and P30. Synapses were stained with the presynaptic anti‐CtBP2 (red) and postsynaptic anti‐GluR2 (green) antibodies, and nuclei were labeled with DAPI (blue). Scale bar = 5 µm. B–D) Quantitative analysis of CtBP2‐positive puncta, GluR2‐positive puncta, and ribbon synapses. The number of GluR2‐positive puncta in the *Dyx1c1^−/−^* mice was higher than that in WT mice at P12 and P18. Data are shown as the mean ± S.D. ***p < 0.001 using two‐tailed, unpaired Student's *t*‐tests.

The abnormal spontaneous activity of IHCs and SGNs in *Dyx1c1^−/−^* mice indicated defective cochlear development. We hypothesized that there was no stable association between IHCs and SGNs and that this resulted in decreased SGN activity. Therefore, we next performed immunofluorescence analysis of ribbon synapses of IHCs by labeling presynaptic and postsynaptic receptors with anti‐CtBP2 and anti‐GluR2 antibodies, respectively (**Figure** **7**A). The number of GluR2‐positive puncta in the *Dyx1c1^−/−^* mice was significantly greater than WT mice at P12, but this number began to decrease at P18 and was similar to WT mice at P30. In addition, CtBP2‐positive puncta and synapse numbers were consistent with WT mice from P12 to P30 (Figure 7B–D). These results suggest that *Dyx1c1* deficiency in the inner ear leads to impaired SGN development and that hearing is not restored despite the completion of SGN branch pruning at P30. According to previous studies, SGNs will gradually degenerate when there is no signal input from HCs.^[^ ^56^, ^57^ ^]^ Surprisingly, the number of SGNs in the cochleae of *Dyx1c1^−/−^* mice was comparable to WT mice at P30 (Figure S3A,B, Supporting Information). These results suggest that despite delayed pruning of SGN terminal branches in the cochlea of *Dyx1c1^−/−^* mice, this developmental disorder still leads to severe hearing impairment.

## 3 Discussion

**Figure 8:**
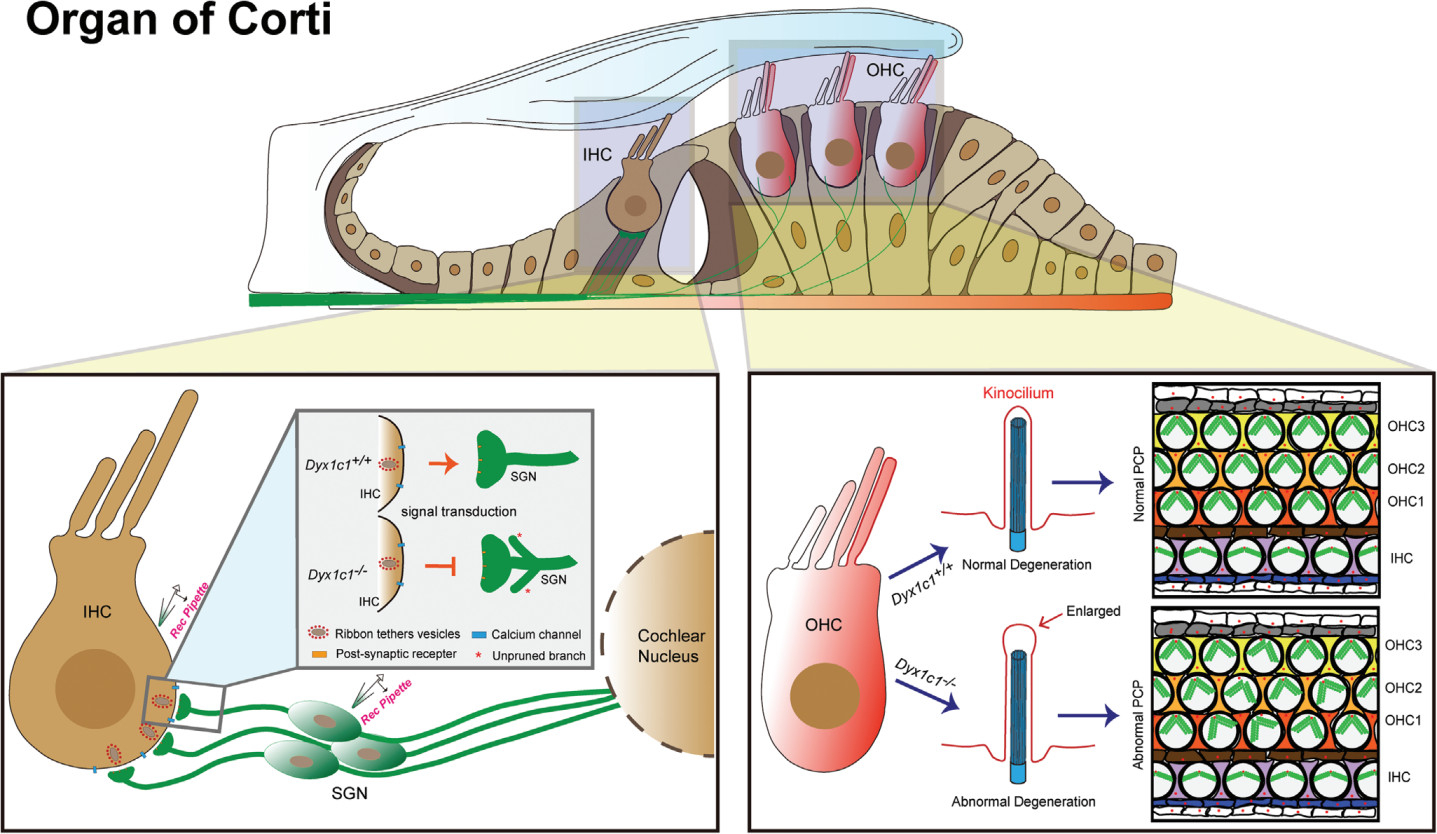
Schematic of abnormal in HCs and type I SGNs caused by *Dyx1c1* knockout. Compared with WT mice, the process of type I SGN refinement and signaling transduction between the IHC and type I SGN is disrupted in the cochlea of *Dyx1c1^−/−^* mice (Bottom left panel). In addition, *Dyx1c1* deficiency damaged the structure of kinocilium and PCP of hair bundle (Bottom left panel). OHC, outer hair cell; IHC, inner hair cell; SGN, spiral ganglion neuron; PCP, planar cell polarity.

To date, a variety of dyslexia‐related pathological deficits have been identified, such as impaired speech processing,^[^ ^58^ ^]^ situs inversus,^[^ ^59^ ^]^ short‐term memory impairment,^[^ ^13^ ^]^ and difficulties with rapid auditory processing.^[^ ^60^ ^]^ In addition, dyslexia patients sometimes suffer from hearing loss, but the mechanism of DRHL is unclear. *Dyx1c1* is the first reported dyslexia candidate gene, and *Dyx1c1^−/−^* mice have become a good model for dyslexia research.^[^ ^33^, ^34^, ^50^, ^61^ ^]^ In addition to dyslexia, *Dyx1c1* deficiency is involved in various biological processes such as neuron migration^[^ ^62^ ^]^ and cilia assembly.^[^ ^63^ ^]^ In this study, we found that *Dyx1c1* is widely expressed in the mouse cochlea, and we used CRISPR‐Cas9 technology to construct *Dyx1c1^−/−^* mice with severe hearing loss. The structure of kinocilia was altered in the cochleae of *Dyx1c1^−/−^* mice, and the PCP of the hair bundle was disrupted, in addition, patch‐clamp experiments showed altered early spontaneous electrical activity in the type I SGNs and IHCs in the cochleae of *Dyx1c1^−/−^* mice (**Figure** **8**), which suggesting a possible mechanism for DRHL. These results suggest that *Dyx1c1* gene deletion causes dyslexia‐related organ positioning defects and that hearing loss in *Dyx1c1* ^−/−^ mice might be due to functional disruption of HCs and SGNs.

The expression level of *Dyx1c1* is high in the tectorial membrane, spiral limbus, etc., and in general molecules that are abundant in cells or tissues are presumed to be involved in key biological processes.^[^ ^64^ ^]^ However, we found that the structures of the tectorial membrane, spiral limbus, etc., did not exhibit obvious defects in the *Dyx1c1^−/−^* mice used in this study, but we cannot rule out that loss of *Dyx1c1* does not alter the biological function of these elements. In this study, the dyslexia candidate gene *Dyx1c1* was shown to be involved in maintaining the structure of the kinocilia and hair bundles in the cochlea. Coincidentally, the dyslexia candidate genes *ROBO1*, *DCDC2*, and *KIAA031* have also been shown to be involved in ciliary function, which indicates that dyslexia is associated with cilia‐related diseases.^[^ ^35^, ^40^, ^41^ ^]^ In addition, abnormal cilia function can lead to severe diseases such as situs inversus, sterility, and neuronal development disorders, which are very similar to the phenotypes associated with dyslexia.^[^ ^35^, ^65^, ^66^, ^67^ ^]^ In this study, the *Dyx1c1* ^−/−^ mice showed situs inversus and ciliary dysfunction similar to PCD, which is consistent with the study of Aarti Tarkar et al.^[^ ^35^ ^]^ Based on these results, we conclude that PCD‐related genes are involved in DRHL.

In this study, *Dyx1c1* ^−/−^ mice exhibited severe hearing loss. We first observed the structural differences of kinocilia and microvilli in *Dyx1c1* ^−/−^ mice at P4, and then found that the PCP of hair cells was disrupted at P0 but recovered at P4. Catherine Copley^[^ ^49^ ^]^ showed that conditional knockout of the Van Gogh‐like 2 (*Vangl2* CKO) in cochlear HCs resulted in dysregulated PCP. However, the dysregulated PCP was repaired by an unknown mechanism, but the mice still have severe hearing loss, the ABR threshold of *Vangl2* CKO mice from 4 to 32 kHz was about 20 dB higher than WT mice. Another study showed that ciliogenesis associated kinase 1 gene (*Cilk1* CKO) in the mouse cochlea disrupted the kinociliary structure and PCP of HCs; however, the *Cilk1* CKO mice had normal hearing at high and medium frequencies and only showed hearing impairment at low frequencies.^[^ ^48^ ^]^ In this study, we surprisingly found that the PCP of hair cells was disrupted in *Dyx1c1* ^−/−^ mice at P0 but subsequently recovered at P4. Via checking a large number of studies related to core PCP proteins, we get the conclusion that even PCP is largely destroyed at the early time, they reoriented and recovered. For example, when *Vangl2* (one of core PCP proteins) was knockout, the PCP of hair bundles was severely disrupted, but it was subsequently reoriented. This phenomenon suggests that even the loss of core PCP proteins is not necessary for the thereafter established hair bundle polarity. The reason which leads to this is still unknown up to now and deserves to explore in the future. These reports suggest that slight alterations of kinocilia structure and PCP in the mouse cochlea do not cause severe hearing impairment as is seen in *Dyx1c1* ^−/−^ mice, and it is predicted that other mechanism leads to severe hearing loss.

Dyslexia has also been reported to be associated with neuronal developmental disorders, and there are a large number of auditory neurons in the cochlea, the functional defects of which can lead to severe hearing loss. In this study, we found that *Dyx1c1* is highly expressed in SGNs, and the latency and amplitude values of ABR peak 1 in *Dyx1c1* ^−/−^ mice suggest that SGN dysfunction may be another important factor leading to hearing loss. We confirmed this hypothesis by examining the physiological activity of SGNs using the patch‐clamp technique. Our results showed that the spontaneous electrical activity of IHCs in *Dyx1c1* ^−/−^ mice was greater than WT mice, but the electrical activity of SGNs was lower than WT mice, indicating that the signal transmission process between IHCs and SGNs was hindered. Spontaneous electrical activity is a common feature during the early development of the sensory system, and early spontaneous action potentials in the SGN contribute to the maturation and stabilization of neural circuits between HCs and the auditory cortex. Thus, the altered spontaneous electrical activity of SGNs in *Dyx1c1* ^−/−^ mice might be a key factor in their severe hearing loss. Taken together, our results suggest that DRHL is due to SGN developmental disorders and cilia defects.

Our study confirms that *Dyx1c1* ^−/−^ mice are a good disease model for studying DRHL. In addition to regulating cilia‐related biological functions in the cochlea, *Dyx1c1* is also essential for the maturation of the SGN. Thus, our findings provide new insights into changes in ciliary function and the early spontaneous electrical activity of SGNs caused by *Dyx1c1* gene deficiency, thus providing a theoretical foundation for the pathogenesis and prevention of DRHL. Overall, our study provides new clues for the clinical diagnosis of DRHL and provides strong evidence that PCD is involved in DRHL. Our study further enriches the understanding of pathogenic mechanism of dyslexia, and suggests that *Dyx1c1* is the first candidate gene associated with DRHL, which may serve as a potential target for clinical diagnosis and treatment of DRHL.

## 4 Experimental Section

### Mice and Genotyping

All procedures were performed according to the research guidelines of the Institutional Animal Care and Use Committee (IACUC) at Southeast University and conformed to the National Institutes of Health Guide for the Care and Use of Laboratory Animals. This work made every effort to minimize the number of animals used and to reduce their suffering. The *Dyx1c1* gene was deleted using CRISPR‐Cas9. For genotyping, mouse tail tissue was obtained for PCR experiments with the following primer sequences: 5′‐CAC CCT GCT TCT ACC TCC‐3′ (WT – Forward), 5′‐GGC TTT GCT CTT CCC ATC ‐3′ (WT – Reverse), 5′‐ TGG AAT AAA CGG TTG TAA‐3′ (Knockout – Forward), and 5′‐AAT CTT GGC TTT GCT CTA‐3′ (Knockout – Reverse).

### Immunofluorescence

The mouse cochlear temporal bone was rapidly dissected in cold PBS and then fixed in 4% PFA overnight at room temperature. The solution was replaced with 0.5 m EDTA the next day to decalcify the temporal bone, and this was incubated for 3 days. The mouse cochlear basement membrane was dissected using a microscope and was adhered to a round glass slide with Cell‐Tak (BD Biosciences, 354 240). The cochlear tissue was immersed in PBST (1% Triton X100 in PBS) solution for 15 min and then incubated in blocking solution (10% goat serum in PBS) at room temperature for 1 h. Cochlear tissue was incubated in PBS containing primary antibody overnight at 4 °C. The next day, the tissue was washed three times for 5 min each in PBST solution and then incubated in PBS solution containing secondary antibody at room temperature for 1 h. Finally, the samples were mounted with DAKO (S3023) solution after washing in PBST. Cochlear samples were imaged under a confocal microscope (Zeiss Lsm 700). All antibody information is in Table S1, Supporting Information.

### ABR Measurement

The ABR threshold is commonly used to assess hearing function. Mice were anesthetized with sodium pentobarbital (100 mg kg^−1^) by intraperitoneal injection. Active electrodes were inserted into the skull base, with a reference electrode located below the tested ear and a ground electrode located near the tail. ABR thresholds of the mice were recorded at five frequencies (4, 8, 12, 16, 24, and 32 kHz) using the BioSigRZ software (TDT, Gainesville, FL, USA) and a Tucker–Davis Technology System III (TDT). Sound pressure level (dB SPL) was measured in 5 dB increments from 10 to 90 dB, and the hearing threshold was defined as the lowest sound pressure level that caused a detectable auditory response. Both female and male mice were tested.

### Western Blot

Mice were sacrificed by cervical dislocation, and the temporal bones were rapidly removed and placed in eppendorf tubes containing a protease inhibitor cocktail (Sigma, 0 469 313 2001) and medium‐strength RIPA lysis buffer (Beyotime, P0013k). Two pre‐cooled magnetic beads were added to the tubes, and the eppendorf tubes were placed in a high‐throughput tissue grinder (Chengk Instruments, Grinder‐48) and ground three times for 1 min with a 1 min interval between each grinding. The tubes were then centrifuged at 14 000× g at 4 °C for 10 min. The supernatant was then mixed with an equal volume of SDS and then boiled in water for 10 min for western blot experiments or stored at −20 °C. Protein samples were soaked in boiling water for 10 min before performing western blotting, and each sample was separated using SDS‐PAGE and then transferred to a PVDF membrane (Millipore, IPVH00010). The membranes were blocked with 5% skim milk in TBST (Beyotime, ST673) for 1 h at room temperature and then incubated with the primary antibody in TBST overnight at 4 °C. The next day, the membrane was washed with PBST three times for 10 min each and then incubated with secondary antibody for 1 h at room temperature and then imaged with ECL reagent (Vazyme, E412‐01).

### Statistical Analysis

Statistical analysis was performed using a two‐tailed Student's *t*‐test, and all results are expressed as the mean ± SD as indicated from at least three independent experiments. All data were analyzed using GraphPad Prism 9.0 (GraphPad Software). For all tests, a *p*‐value < 0.05 was considered statistically significant.

## Conflict of Interest

The authors declare that there are no conflicts of interest.

## Author Contributions

G.H., X.F., X.C., and L.Z., contributed equally to this work. G.H. and R.C. designed the project. G.H., X.F., X.C., L.Z., X.H., Z.L., X.B., W.L., S.D., M.C., and H.T. performed the experiments and acquired the data. G.H., R.C., and X.F. analyzed the results and wrote the manuscript.

## Supporting information

Supporting Information

## Data Availability

The data that support the findings of this study are available from the corresponding author upon reasonable request.
