# Correction to “Dyslexia‐Related Hearing Loss Occurs Mainly Through the Abnormal Spontaneous Electrical Activity of Spiral Ganglion Neurons”

**DOI:** 10.1002/advs.202413887

**Published:** 2024-12-04

Guodong Hong, Xiaolong Fu, Xin Chen, Liyan Zhang, Xuan Han, Shuqin Ding, Ziyi Liu, Xiuli Bi, Wen Li, Miao Chang, Ruifeng Qiao, Siwei Guo, Hailong Tu, Renjie Chai

*Adv. Sci*. 2023, 10, e2205754.

https://doi.org/10.1002/advs.202205754

In the original published paper, we found that the images of the WT group in Figure 4A and the “Gapdh” band in Figure 6H were improperly used. The corrected figures are shown below.

Corrected Figure 4A:


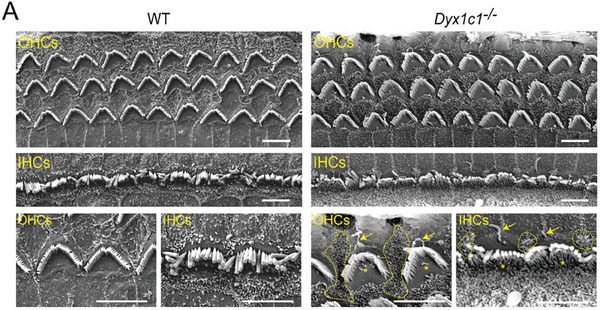


Corrected Figure 6H:


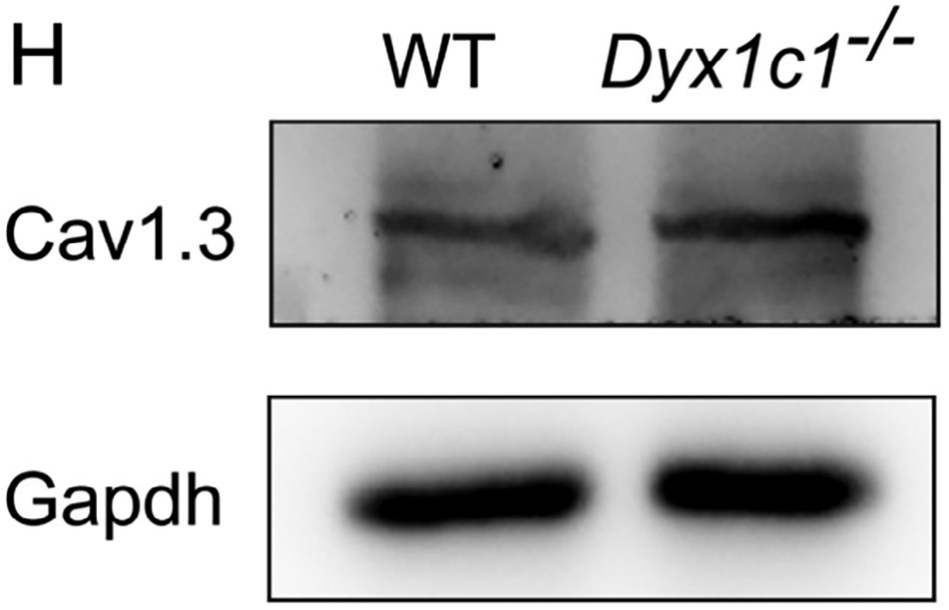


In Figure S2A (Supporting Information), the order of the “Apical” and “Middle” images of the P4 *Dyx1c1^−/‐^* group was used incorrectly. The corrected figure is shown below.

Corrected Figure S2A (Supporting Information):


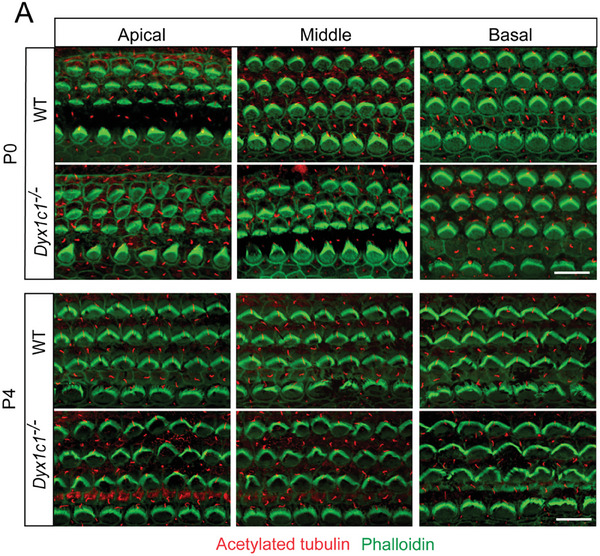


This correction does not affect the overall findings and conclusions of this paper. We apologize for these errors.

