# Supplementary material for: Dyslexia‐Related Hearing Loss Occurs Mainly through the Abnormal Spontaneous Electrical Activity of Spiral Ganglion Neurons
Source: Adv Sci (Weinh). 2023 Apr 17;10(16):2205754. doi: 10.1002/advs.202205754 (PMC10238183; doi:10.1002/advs.202205754)
Supplement: Supplementary file 1 — Supporting Information [file ADVS-10-2205754-s001.pdf]

Supplementary Figure 1. Analysis of the NOR and representative ABR waveforms of *Dyx1c1*$^{-/-}$ mice.

(A) NOR analysis showed that the *Dyx1c1*$^{-/-}$ mice had poor recognition memory for novel objects. n = 3 for each group. **(B)** ABR waveforms at 16 kHz in P60 WT and *Dyx1c1*$^{-/-}$ mice. The red line represents the minimum hearing threshold that mice could perceive at this frequency. Data are shown as mean ± S.D. *$p < 0.05$ using two-tailed, unpaired Student's t-tests.

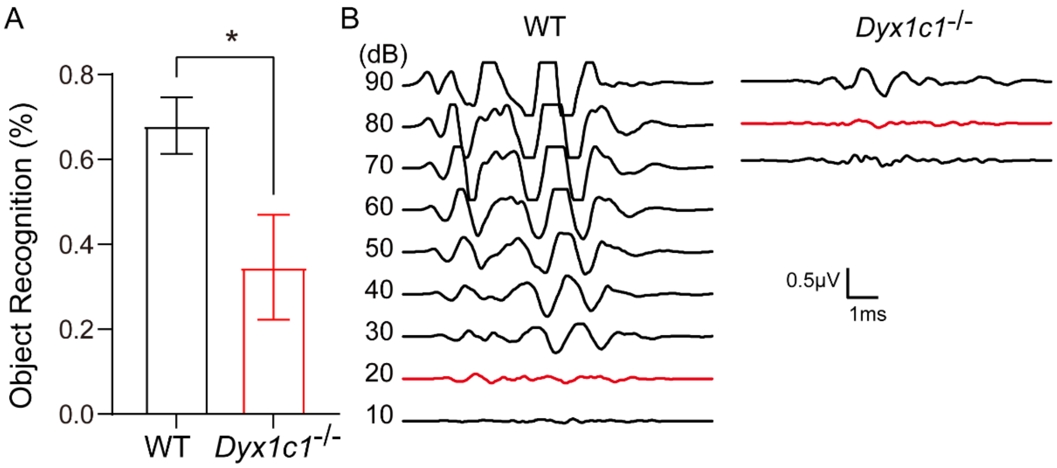

Supplementary Figure 2. Cochlear HC PCP was disrupted in early postnatal *Dyx1c1*$^{-/-}$ mice.

(A) Immunofluorescence staining of the basement membrane of P0 and P4 WT and *Dyx1c1*$^{-/-}$ mice. Kinocilia and hair bundles were labeled with anti-acetylated tubulin (red) and phalloidin (green) antibodies, respectively. Scale bar = 10 μm. **(B)** Schematic illustration of hair bundle deflection. The red dots represent the kinocilia, and the green areas represent hair bundles. **(C)** In WT mice, every hair bundle was oriented from the neural towards the abneural side of the sensory epithelium. **(D, E)** Compared with WT mice at P0, *Dyx1c1*$^{-/-}$ mice had disrupted PCP of hair cells in the apical and middle turns of the cochlea, and PCP deficiency was more severe in OHCs than IHCs. ***$p < 0.001$ using two-tailed, unpaired Student's t-tests.

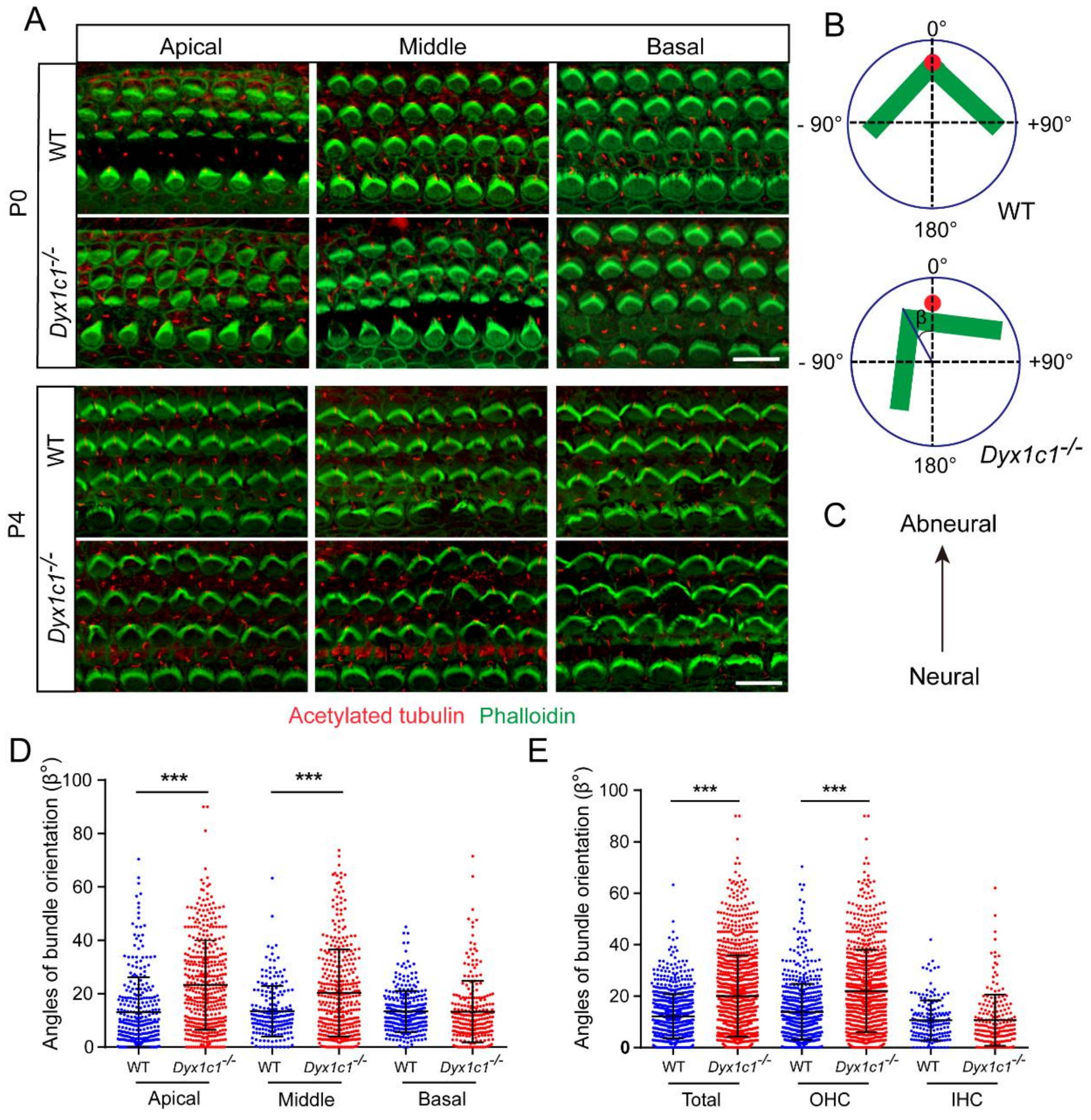

Supplementary Figure 3. There was no significant change in the number of SGNs at P30.

(A) Immunofluorescence staining after cryosectioning of cochleae from P30 WT and *Dyx1c1*$^{-/-}$ mice. Anti-Tuj1 antibody was used to label SGNs (red), and DAPI was used to label the nuclei (blue). Scale bar = 50 μm. **(B)** Quantitative analysis of SGNs in (A).

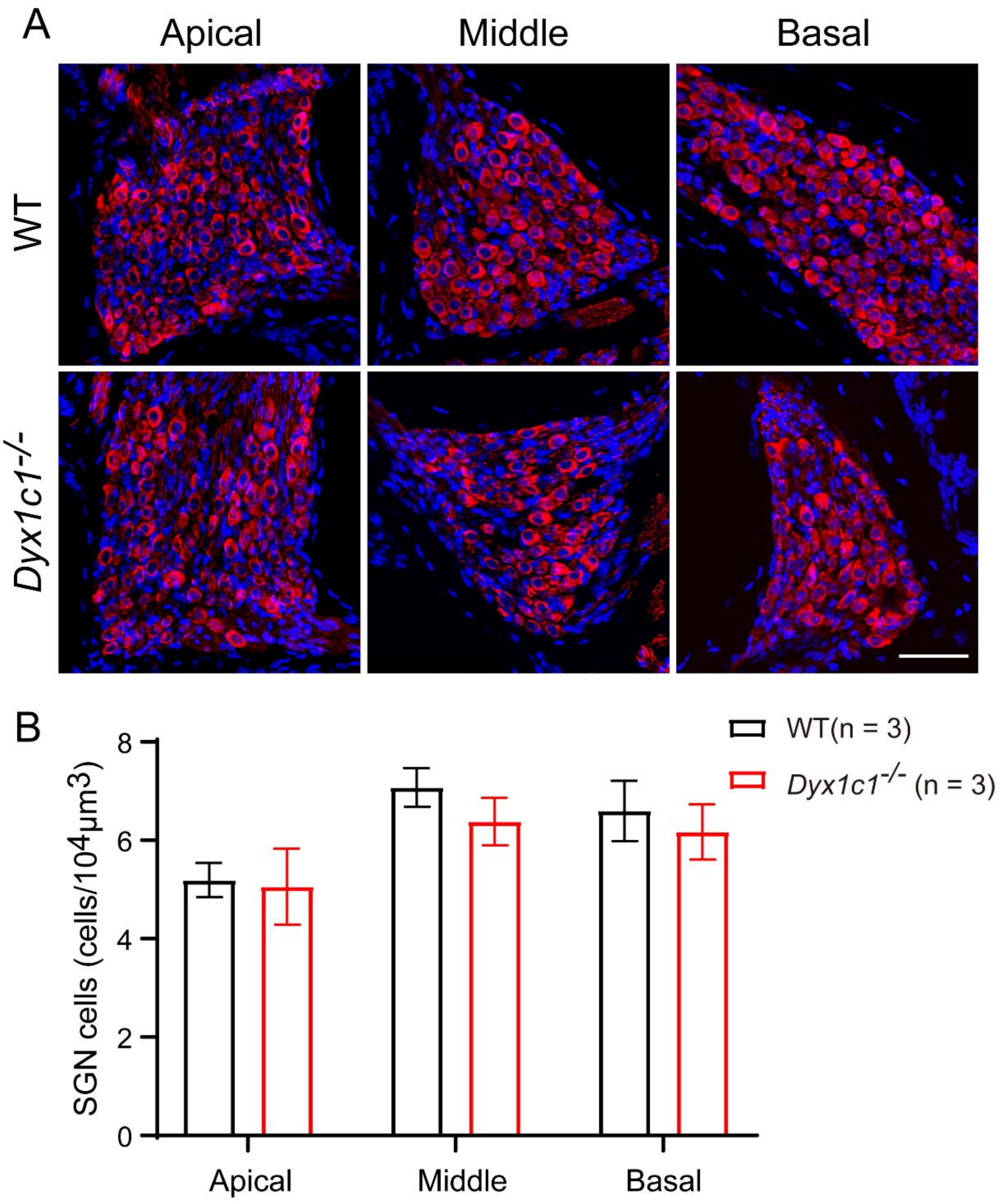

Supplementary Table 1. Summary of antibody information.

Antibody	Source	Cat#	Application
Anti- DYX1C1	Proteintech	14522-1-AP	IF/WB
Anti-GAPDH	Protein Biotechnologies	PMC203	WB
Anti-Myosin7a	Proteus Bioscience	25-6790	IF
Anti-Myosin7a	DSHB	138-1	IF
Anti-Sox2	Santa Cruz	Sc-17320	IF
Anti-Prestin	Santa Cruz	Sc-22692	IF

Anti-vGlut3	Synaptic Systems	135203	IF
Anti-Cav1.3	Allomone labs	ACC-005	WB
Anti-Acetylated Tubulin	Proteintech	66200-1-Ig	IF
Anti-IFT88	Proteintech	13967-1-AP	IF/WB
Anti-Tuj1	Abcam	Ab18207	IF
Anti-GluR2	Merck Millipore	MAB397	IF
Anti-CtBP2	BD Biosciences	612044	IF

IF: immunofluorescence; WB: Western blot
